# Climatic Refugia and Geographical Isolation Contribute to the Speciation and Genetic Divergence in Himalayan-Hengduan Tree Peonies (*Paeonia delavayi* and *Paeonia ludlowii*)

**DOI:** 10.3389/fgene.2020.595334

**Published:** 2021-01-27

**Authors:** Yu-Juan Zhao, Gen-Shen Yin, Yue-Zhi Pan, Bo Tian, Xun Gong

**Affiliations:** ^1^Key Laboratory for Plant Diversity and Biogeography of East Asia, Kunming Institute of Botany, Chinese Academy of Sciences (CAS), Kunming, China; ^2^Key Laboratory of Economic Plants and Biotechnology, Kunming Institute of Botany, Chinese Academy of Sciences (CAS), Kunming, China; ^3^College of Agriculture and Life Sciences, Kunming University, Chinese Academy of Sciences (CAS), Kunming, China; ^4^Key Laboratory of Tropical Plant Resource and Sustainable Use, Xishuangbanna Tropical Botanical Garden, Chinese Academy of Sciences, Mengla, China

**Keywords:** climate change, Himalaya-Hengduan Mountains, *Paeonia*, Pleistocene, refugia, speciation

## Abstract

Himalaya and Hengduan Mountains (HHM) is a biodiversity hotspot, and very rich in endemic species. Previous phylogeographical studies proposed different hypotheses (vicariance and climate-driven speciation) in explaining diversification and the observed pattern of extant biodiversity, but it is likely that taxa are forming in this area in species-specific ways. Here, we reexplored the phylogenetic relationship and tested the corresponding hypotheses within *Paeonia* subsect. *Delavayanae* composed of one widespread species (*Paeonia delavayi*) and the other geographically confined species (*Paeonia ludlowii*). We gathered genetic variation data at three chloroplast DNA fragments and one nuclear gene from 335 individuals of 34 populations sampled from HHM. We performed a combination of population genetic summary statistics, isolation-with-migration divergence models, isolation by environment, and demographic history analyses. We found evidence for the current taxonomic treatment that *P*. *ludlowii* and *P*. *delavayi* are two different species with significant genetic differentiation. The significant isolation by environment was revealed within all sampled populations but genetic distances only explained by geographical distances within *P*. *delavayi* populations. The results of population divergence models and demographic history analyses indicated a progenitor–derivative relationship and the Late Quaternary divergence without gene flow between them. The coalescence of all sampled cpDNA haplotypes could date to the Late Miocene, and *P*. *delavayi* populations probably underwent a severe bottleneck in population size during the last glacial period. Genetic variation in *Paeonia* subsect. *Delavayanae* is associated with geographical and environmental distances. These findings point to the importance of geological and climatic changes as causes of the speciation event and lineage diversification within *Paeonia* subsect. *Delavayanae*.

## Introduction

Mountains are often hotspots of endemism, where the geological activities and climatic changes are historically frequent, driving lineage divergence, and speciation both in tropical and temperate regions (Myers et al., 2000; Perrigo et al., 2020). Himalaya-Hengduan Mountains (HHM) are biodiversity hotspots (Myers et al., 2000; Lopez-Pujol et al., 2011), located to the east and south of the Qinghai-Tibetan Plateau (QTP). It has been suggested that the uplift of QTP began 40–50 million years ago (Ma) and underwent final extension during the Late Miocene (c. 10 Ma), and Hengduan Mountains (HDM) experienced major uplift after the Miocene and reached peak elevation shortly before the Late Pliocene, resulting in parallel north–south-oriented valleys surrounded by high peaks (Favre et al., 2015; Xing and Ree, 2017). Climatic factors are often as dynamic as topography, and climatic changes over space and time may promote speciation via niche conservatism or niche divergence (Hua and Wiens, 2013). Moreover, it is currently recognized that the climatic evolution during the Quaternary coupled with the effects of complex tectonic events has largely shaped the species richness in this area, in terms of both the presence of species with broad distribution and aggregation of locally endemic taxa (Lopez-Pujol et al., 2011; Xing and Ree, 2017; Yu et al., 2019).

Disentangling the relative roles of geographical and ecological factors as drivers of interspecific and intraspecific diversification has been challenging but is of central importance in explaining biogeographical patterns and speciation processes (Warren et al., 2014). If allopatric speciation associated with geographical isolation was the dominant mode of lineage differentiation or speciation, we would expect most sister–species pairs to be allopatrically distributed, but they might show little ecological niche differentiation because this would not be needed for speciation to occur, and new species tends to keep the ancestor niche (i.e., niche conservatism; Wiens and Graham, 2005). However, when the divergence pace of lineage and niche exhibits consistency, ecological niche divergence may also have contributed to speciation, regardless of the geographical mode of speciation (Hua and Wiens, 2013). During the last decades, genetic effects of vicariant events induced by topographical change are well-documented in phylogeographic studies, highlighting the existence of cryptic diversity in many species such as yews, oaks, moles, and white pine distributed in HHM (Liu et al., 2013, 2019; Du et al., 2017; He et al., 2019). However, comparatively little is known about the potential role of ecological factors in the process of species or lineage diversification, and ecology-driven genetic divergence has seldom been examined (Meng et al., 2015; Zhao et al., 2016; Gao et al., 2019).

*Paeonia* L. (Paeoniaceae), is a monotypic genus with a long evolutionary history and possesses germplasm resources with significant medicinal values (Dong et al., 2018). However, most of these tree peonies have become vulnerable or endangered with narrowly restricted populations having small numbers of individuals due to human overexploitation and domestication (Hong et al., 2017). *P*. *delavayi*, belonging to subsect. *Delavayanae*, is the only non-endangered one with widespread distribution area in northern Yunnan, western Sichuan, and southeastern Tibet and occurring at a range of altitudes (c. 1,900–4,000 m). Moreover, great morphological diversity exists within *P*. *delavayi*, and this diversity was previously ascribed to several species (such as *Paeonia lutea, Paeonia potaninii*, and *Paeonia trollioides*), They exhibited continuous and overlapping variation in leaf and flower color characters without geographic patterns and then thus amalgamated into one species *P*. *delavayi* (Hong et al., 1998). *Paeonia ludlowii* (Stern & Taylor) D. Y. Hong, the other member of subsect. *Delavayanae*, is geographically confined to southeastern Tibet with a high average but narrow altitude range (c. 2,920–3,320 m). *P*. *ludlowii* was firstly reported as one variety of *P. lutea* by Stern and Taylor (1951) and elevated to species rank based on a suite of distinguished characters: a height of 1.5–3.5 m, pure yellow petals, and 1–2 carpels (*P*. *ludlowii*) (Hong, 1997; Hong and Pan, 2005).

Currently, there is some consensus about the taxonomy of subsect. *Delavayanae*, but the genetic boundary between the two species is still controversial (Zhang J. M. et al., 2018). However, caution against these analyses based on limited sample sizes should be considered for species delimitation (Zhang et al., 2009; Zhou et al., 2014), and proper samplings (e.g., at least 10 samples) ensuring that the most meaningful genetic variation is sampled have been suggested (Carstens et al., 2013). In addition, although three clusters coexist across Himalaya (*P*. *ludlowii*), Tibetan, and the rest populations of *P*. *delavayi* based on the SSR dataset (Zhang J. M. et al., 2018), whether the environment is independent from geographical isolation creating genetic divergence within and between the species of subsect. *Delavayanae* remains poorly understood.

Here, we build on previous studies by analyzing a more extensive genetic dataset (in terms of both the number of individuals and populations and the different nuclear gene markers), to test the hypothesis on geology- vs. climate-driven divergence within subsect. *Delavayanae*. If Pleistocene climate oscillations did trigger speciation events, we expect that the divergence would date to the major glacial cycles and environment isolation perhaps will also be responsible for the lineage differentiation. Specifically, we reconstruct the phylogenetic relationship in subsect. *Delavayanae*, test the relative contribution of geography and environment in shaping the pattern of genetic variation within and between the two species, and explore the demographic history to elucidate divergence process and possible gene flow between the two species. Our study will contribute to a better understanding of the role played by tectonic events and Quaternary climate change on the lineage divergence and speciation of organisms in HHM.

## Materials and Methods

### Sample Collection

From 2015 to 2018, a total of 34 populations were sampled in China. *P*. *delavayi* were collected from 31 locations covered the geographic distribution range (YP: Yunnan Plateau, HDM: Hengduan Mountains, and EH: East Himalaya) and three populations of *P*. *ludlowii* were sampled to represent the geographical spread in Mainling, Tibet (EH) (Supplementary Table 1). Within each population of *P*. *delavayi*, individuals growing at least 20 m were collected to minimize the impacts of vegetative reproduction by rhizomes. Sampling was restricted to adults and defined by the presence of flowers or fruits. Samples of *Paeonia ostii* and *Paeonia mairei* were used as outgroups.

### DNA Extraction, Chloroplast DNA, and Nuclear Gene Sequencing

Genomic DNA was extracted from the silica gel-dried leaves using the CTAB method with some modifications (Doyle, 1991). Three chloroplast DNA fragments (*trn*L-*trn*F, *rps*16-*trn*K, *trn*H-*psb*A) and one presumably single-copy nuclear gene (ATP-dependent Lon protease) were amplified and sequenced using the published primers (Supplementary Table 2). Gametic phases for nuclear genes were determined using PHASE in DnaSP 5.0 (Librado and Rozas, 2009), and individuals that could not be directly sequenced were cloned.

### Population Structure Analyses

We aligned DNA sequences with ClustalX and manually checked with Bioedit. Haplotypes were defined using DnaSP 5.0 (Librado and Rozas, 2009), and a media-joining network was calculated under default parameters using NETWORK (Bandelt et al., 1999). Genetic differentiation between the two species and among populations within each species was examined using hierarchical AMOVAs in Arlequin 3.5 (Excoffier and Lischer, 2010) with significance tested using 1,000 permutations. To test for genetic structures within and between sampling locations, we also conducted analyses using STRUCTURE 2.3 (Pritchard et al., 2000). We assumed that individuals followed the admixture model in STRUCTURE and that allele frequencies were correlated among groups. STRUCTURE was run using a 50,000 burn-in and 5,00,000 Markov chain Monte Carlo (MCMC) for potential K of 1–30 for 10 replicates each. The optimal clustering number (K) was determined by calculating the mean L(K) and ΔK (Evanno et al., 2005) using STRUCTURE HARVESTER (Earl and Vonholdt, 2012). Then, CLUMPP (Jakobsson and Rosenberg, 2007) was used to average each individual's admixture proportions over the 10 replicates for the best K, and those results were input into DISTRUCT (Rosenberg, 2004), producing a summary image of the runs.

### Phylogenetic and Biogeographic Analyses

We used the simple insertion/deletion (indels) coding method in Gapcoder to code all contiguous indel events involving more than one base pair as one mutation event (Simmons and Ochoterena, 2000). The phylogenetic relationships among nuclear gene haplotypes was reconstructed using maximum likelihood (ML) in RAxML (Stamatakis, 2006) and Bayesian inference (BI) in MrBayes (Ronquist and Huelsenbeck, 2003). The default GTR nucleotide substitution model was used in ML analyses. The best substitution model selected from jModelTest 2.1.5 (Darriba et al., 2012) using the Akaike information criterion (AIC) was used in Bayesian analysis. We assessed the tree support with a bootstrap (BS) analysis with 10,000 replicates for ML and posterior probabilities (PP) with 15 million generations for BI, respectively.

Time to most recent common ancestor (TMRCA) of cpDNA haplotypes was estimated in BEAST 1.8.2 (Drummond and Rambaut, 2007). For the lack of peony fossils, the cpDNA mutation rate of 3.18 × 10^−10^ substitution per site per year was used. This rate was recently reported by Qi et al. (2012) based on two fossils of Cercidiphyllaceae and Altingiaceae, which have close relationships with Paeoniaceae within Saxifragales (Jian et al., 2008). The MCMC chain was run for 10^7^ generations, sampling parameter values and trees every 1000th generation, following a burn-in of the initial 10% samples. Trees and parameters from the three independent runs were combined to obtain a summary tree using LogCombiner and TreeAnnotator from the BEAST package.

### IBD and IBE Analyses

To evaluate the relative role of environment and geography on population genetic differentiation, we used multiple matrix regression with randomization (MMRR) analysis implemented in R (Wang, 2013). Pairwise *F*st between populations on the basis of nuclear genes were calculated in Arlequin and used as dependent variables. Euclidean geographic distances (Standard) were estimated using the coordinate information in GenAlex (Peakall and Smouse, 2006). We generated the environmental distance matrix using 8 bioclimatic variables among 19 downloaded from the Worldclim database (https://worldclim.org/) at 2.5′ spatial resolution, after removing variables with Pearson's correlation values (|*r*|) >0.9 to reduce multicollinearity, according to Zhang J. M. et al. (2018). Briefly, we extracted the values for each environmental variable at every locality and performed principal component analysis (PCA). The mean scores along the first three components explained most of the environmental variance (94%) and were used for calculating the Euclidean distances between populations. Both PCA and measures of Euclidean distances (environmental distances) were performed in R using “prcomp” and “ecodist” functions, respectively. The matrixes of geographic and environmental distances between populations were used as explanatory variables. The simulations were conducted with 10,000 permutations. All tests above were performed on two different datasets, one containing all sampled populations and the other containing only *P*. *delavayi* populations. In addition, the mean values and standard errors of 8 bioclimatic variables for populations of the HDM, Yunnan Plateau, and Tibet regions were calculated with SPSS and were further analyzed using independent-sample *t*-tests to validate the impact of the climatic factors on the genetic differentiation among the three regions.

### Demographic History Inference

Analyses of historical demographic parameters were firstly conducted using IMa (Hey and Nielsen, 2007) with the combined cpDNA sequences and nuclear datasets. The longest non-recombining regions for the nuclear gene obtained from IMgc analyses (Woerner et al., 2007) were used. IMa estimates six demographic parameters for *P*. *delavayi* and *P*. *ludlowii* species pairs: two effective population sizes for *P*. *delavayi* (θ_1_) and *P*. *ludlowii* (θ_2_), one ancestral effective population size (θ_A_), two bidirectional migration rates (*m*_1_ and *m*_2_), and the splitting time between the two species (*t*). The HKY model was chosen for both cpDNA and nuclear loci. Initial pilot runs were used to select upper bounds on Bayesian priors. The MCMC analyses contained 20,000,000 steps after a burn-in period of 2,000,000 steps. All runs were repeated three times, and results were found to be consistent between runs with random starting seeds. All resultant ESS values were > 200. Parameter estimates were converted to demographic values using 3.18 × 10^−10^ as above. For the average substitution rates of plants which are generally about 4–5 times higher in nDNA than in cpDNA (Wolfe et al., 1987), 1.59 × 10^−9^ of the nuclear gene was assumed, and this is close to those of other nuclear genes using species such as oaks and Juglans with a long evolutionary history (Gugger et al., 2013; Bai et al., 2018). The generation time for the two species was set to be 10 years according to that generally assumed for *Paeonia* species due to their common vegetative reproduction by rhizomes (Xu et al., 2019). Secondly, we used the Extended Bayesian Skyline Plot (EBSP) implemented in BEAST to assess historical changes in effective population size, which could incorporate data from multiple independent loci and has the advantage of not assuming any a priori demographic scenario (Heled and Drummond, 2008). We selected the model of nucleotide substitution using jModelTest 2.1.5. We used the same substitution rates for the cpDNA and nuclear gene as above and a strict clock model. All results were analyzed with Tracer 1.5 (Rambaut et al., 2009).

Finally, to explore the demographic history of *Paeonia* subsect. *Delavayanae* further, we also applied the approximate Bayesian computation (ABC) statistical framework to all cpDNA and nuclear data, to compare four scenarios of divergence and population size changes. Scenarios were built considering the IMa and phylogenetic analyses, which point to a possible progenitor–derivative relationship between *P*. *delavayi* and *P*. *ludlowii*. The first scenario consisted of an older and simultaneous divergence of the two species due to the uplifts of the HDM and *P*. *ludlowii* experienced bottlenecks during the glacial period. The second scenario predicts that *P*. *ludlowii* is derived from *P*. *delavayi* during the last glaciation and established its current distribution through recent expansion. Because the distributions of organelle DNA haplotypes and EBSP analysis indicated that *P*. *delavayi* might have experienced a recent bottleneck, in scenarios 3 and 4, population size changes of *P*. *delavayi* were assumed and added on the basis of the first two scenarios. Simulations for each scenario and ABC analyses were conducted using DIYABC (Cornuet et al., 2014). The summary statistics within populations included the number of segregating sites, private segregating sites, variance of pairwise difference, and Tajima's D. Statistics computed between populations were the mean of pairwise differences (W) and Fst between two samples. We generated 0.1 million simulated data sets per scenario. Pre-evaluation of each scenario was performed by PCA, and to identify the best scenario, we used 1% of the simulated data sets closest to the observed data to estimate the relative posterior probability as well as 95% credible interval (95% CI) of each scenario via logistic regression and posterior parameter distributions. The goodness of fit of the scenario was assessed by the option “model checking” with PCA to compare summary statistics between observed and simulated data sets.

## Results

### Sequence Alignment and Haplotype Resolution

The cpDNA alignment consisted of 335 sequences of 2,500 bp yielding 33 haplotypes defined by 35 nucleotide substitutions, 26 indels, and one inversion within the combined three fragments (Supplementary Table 3). Three haplotypes (C17, C27, and C30) were exclusive to *P*. *ludlowii*, and all the rest were found in *P*. *delavayi* (Supplementary Table 1 and Supplementary Figure 1). Almost each population was fixed by one rare haplotype and did not share haplotypes, even between sites such as Pd7, Pd8, and Pd10 distributed in Yulong Mountain belonging to the HDM region.

**Figure 1:**
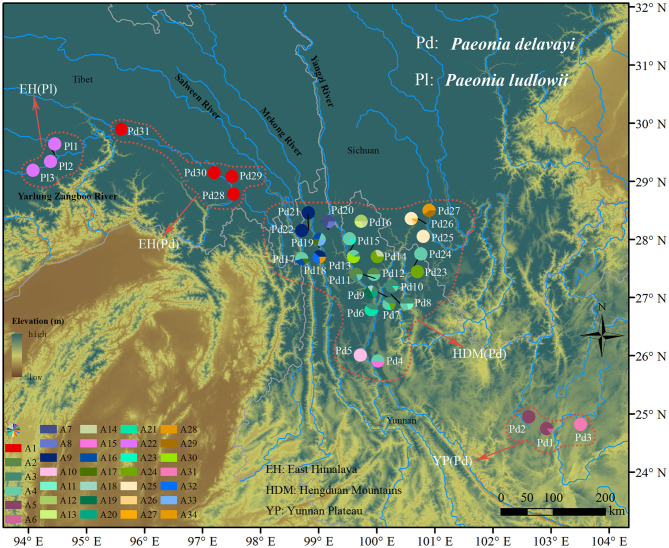
Geographic distribution of 34 nDNA haplotypes recovered from 34 *Paeonia* subsect. *Delavayanae* populations. Pie chart division corresponds to the frequency of haplotype per sampling site. Colored dotted line delineates the population division representing significantly genetic divergence.

For the nuclear dataset, we obtained 670 sequences with a length of 635 bp after being phased in DnaSP, and the aligned sequences distinguished 34 haplotypes defined by 39 nucleotide substitutions and three indels (Supplementary Table 4). Only one haplotype (A22) was found in three populations of *P*. *ludlowii*, while the others were exclusive to *P*. *delavayi*. In HDM, the most common haplotype (A4) was widely distributed and shared by lots of populations, and a high number of private haplotypes were also detected. Populations in EH were fixed by one haplotype (A1), and rare haplotypes (A5, A6, and A31) were found in populations in YP (Supplementary Table 1 and Figure 1).

### Genetic Structure Within and Between Species

**Figure 2:**
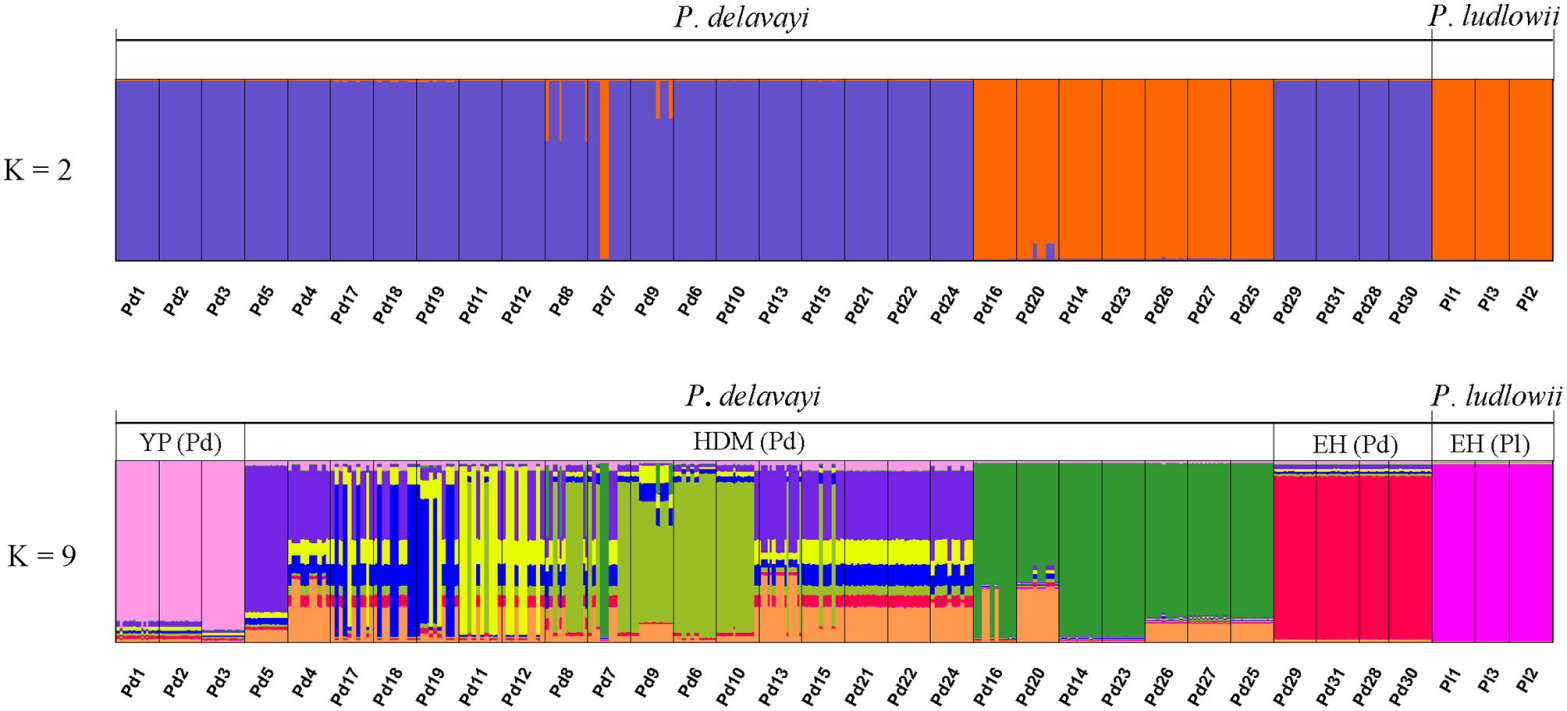
Results of STRUCTURE analyses. Individual assignment plots for *K* = 2 and *K* = 9. When *K* = 9, YP (Pd), HDM (Pd), and EH (Pd) represent three clear groups for *Paeonia delavayi*, and all the populations of *Paeonia ludlowii* were clustered into one group EH (Pl). YP, Yunnan Plateau; HDM, Hengduan Mountains; EH, East Himalaya; Pd, *Paeonia delavayi*; Pl, *Paeonia ludlowii*.

Hierarchical structuring of genetic variation provided by AMOVA revealed that most of the cpDNA genetic variation was found among populations (78.93%) rather than between *P*. *delavayi* and *P*. *ludlowii* (20.77%), whereas nuclear genes showed that the majority of variation (59.07%) separated *P*. *delavayi* and *P*. *ludlowii*, and 37.50% of variation was among populations within species (Table 1). On the species level, 99.71 and 78.90% of the cpDNA variation occurred among *P*. *delavayi* and *P*. *ludlowii* populations, while only 0.29 and 21.10% of the variance were found within *P*. *delavayi* and *P*. *ludlowii* populations, respectively. A significantly high level of genetic differentiation was also detected in the nuclear gene of *P*. *delavayi* (Table 1). Our analysis of the population structure revealed a pattern similar to that inferred from the SSR molecular marker (Zhang J. M. et al., 2018). The delta K (ΔK) results peaked sharply *K* = 2, indicating two distinct genetic clusters but which did not correspond to the two named species (*P*. *delavayi* and *P*. *ludlowii*) (Supplementary Figure 2). The mean L(K) increased significantly after *K* = 2 (Supplementary Figure 2) and gradually became inflated after *K* = 9, in which a highly stable population structure was found and biologically meaningful, with 10 simulations showing the same pattern. For *K* = 9, the two species have their own clusters, and in *P*. *delavayi*, populations from YP, HDM, and EH formed three clear clusters but the genetic composition of HDM samples was almost admixed except for several marginal populations such as Pd25, Pd26, and Pd27 (Figure 2).

**Table 1:** Analysis of molecular variance (AMOVA) of *Paeonia delavayi* and *Paeonia ludlowii* based on cpDNA and nDNA data sets.

**Source of variation**	**cpDNA**					**nDNA**				
	**d.f**.	**Sum of squares**	**Variance components**	**Percentage of variation**	**Fixation indices**	**d.f**.	**Sum of squares**	**Variance components**	**Percentage of variation**	**Fixation indices**
***P***. ***delavayi*** **and** ***P***. ***ludlowii***										
Between species	1	476.134	5.453	20.77	*F*_CT_ = 0.208^*^	1	691.349	6.027	59.07	*F*_CT_ = 0.591^**^
Among populations within species	32	6542.324	20.719	78.93	*F*_SC_ = 0.996^**^	32	2426.102	3.825	37.50	*F*_SC_ = 0.916^**^
Within populations	301	7042.358	0.079	0.30	*F*_ST_ = 0.997^**^	636	222.400	0.350	3.43	*F*_ST_ = 0.965^**^
***P***. ***delavayi***										
Among populations	30	6525.181	21.959	0.997	*F*_ST_ = 0.997^**^	30	2426.102	4.064	0.914	*F*_ST_ = 0.914^**^
Within populations	276	17.900	0.065	0.29		583	222.400	0.381	0.086	
***P***. ***ludlowii***										
Among populations	2	17.143	0.897	0.789	*F*_ST_ = 0.789^**^		–	–	–	–
Within populations	25	6.000	0.240	0.211			–	–	–	–

*d.f., degrees of freedom*,

* P < 0.05 and

** *P < 0.001*.

### Association of Genetic Variation With Geography and Climate

The PCA results showed that the genetic distance of *P*. *delavayi* populations was significantly correlated with the geographical distance (IBD) but not the environmental distance (β_D_ = 0.456, *P* < 0.001 vs. β_E_ = 0.176, *P* = 0.070). However, among the three regions of *P*. *delavayi* (YP, HDM, and EH), the majority of the bioclimatic variables were significantly distinct (Supplementary Table 5). Interestingly, when coupling all the 31 *P*. *delavayi* populations with the three populations of *P*. *ludlowii*, the result displayed a significant correlation between genetic distance and environmental distance (IBE: β_E_ = 0.183, *P* < 0.05), though comparatively geographical distance still contributed more to the total genetic variation (IBD: β_D_ = 0.422, *P* < 0.001).

### Phylogenetic and Phylogeographic Patterns

**Figure 3:**
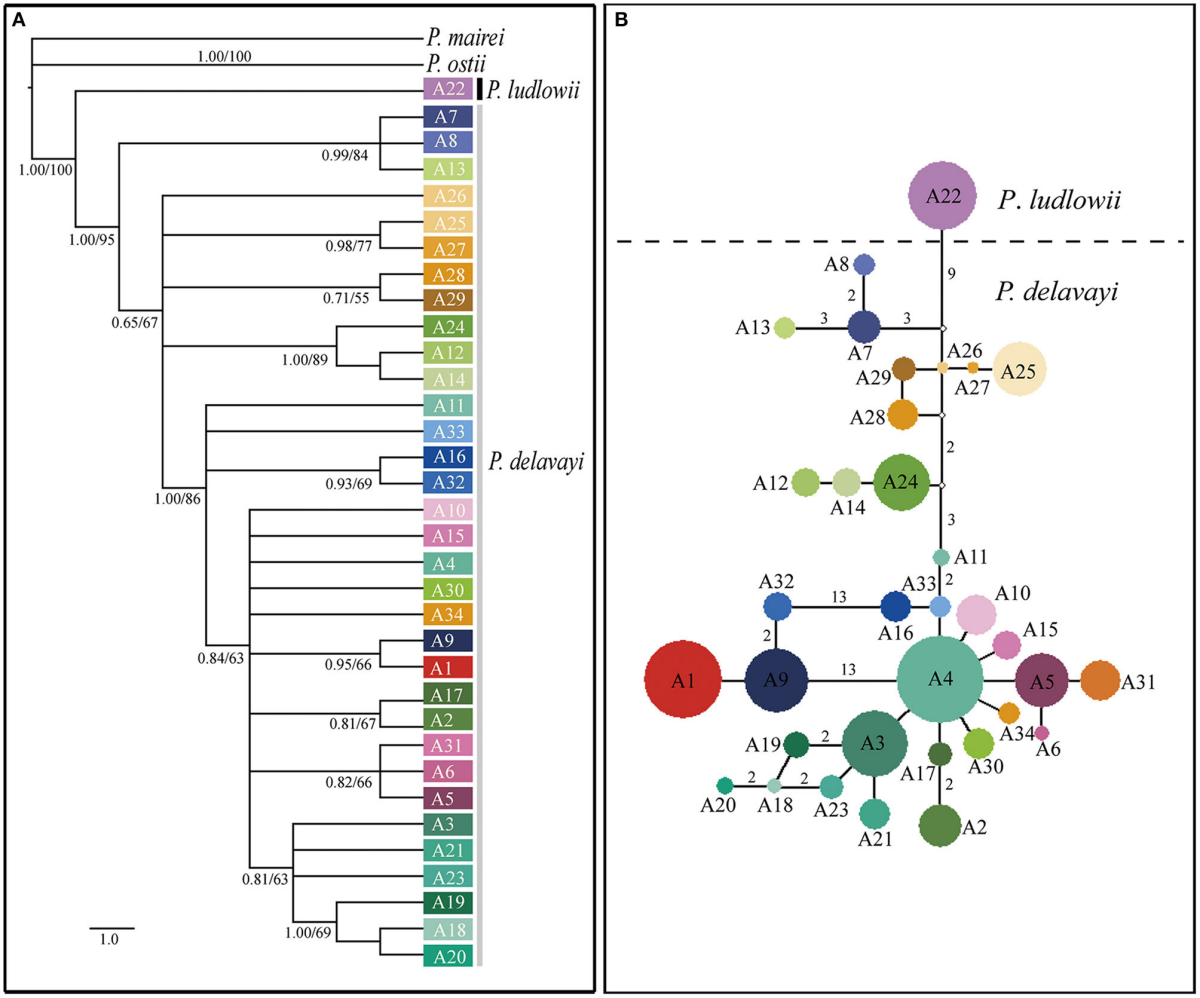
Relationships among the nuclear gene haplotypes of *Paeonia* subsect. *Delavayanae*. **(A)** Phylogenetic tree derived from Bayesian inference. Posterior probabilities and bootstrap support of main clades are shown. **(B)** Haplotype network inferred from NETWORK. The small open circles represent missing haplotype. The sizes of circles are approximately proportional to sample size. Numbers on branches indicate the number of mutations when branches represent more than one mutation.

Phylogenetic trees constructed on the basis of the combined cpDNA sequences showed that *P*. *ludlowii* formed a monophyletic clade nested within *P*. *delavayi* haplotypes, giving limited support for monophyletic groups with subsect. *Delavayanae* (Supplementary Figure 3). By contrast, strong support (89% and 1.00 bootstrap support and posterior probability, respectively) was found for a sister relationship inferred from the nuclear gene tree between *P*. *ludlowii* and *P*. *delavayi*, suggesting a clear genetic boundary between the two currently recognized species. For *P*. *delavayi*, despite strong genetic differentiation, the phylogeny was poorly resolved in these analyses, and no major groupings but numerous paraphyly were detected (Figure 3A and Supplementary Figure 3).

The median joining haplotype network of nuclear gene showed that no haplotype was shared by samples from the three geographical regions (YP, HDM, and EH). Most of major haplotype clades (formed by one or more haplotypes) depicted in the network were roughly concordant with the geographical distribution of populations (Figures 1, 2). The network constructed using cpDNA haplotypes was roughly concordant with that of nuclear genes but showed that a high number (14) of interior missing haplotypes (extinct or not found) were inferred to connect all sampled haplotypes almost in tip positions (Supplementary Figure 1). The Bayesian TMRCA analysis indicated that all sampled cpDNA haplotypes coalesce at about 10.93 Mya (95% HPD: 7.14–15.06 Mya).

### Demographic History

**Figure 4:**
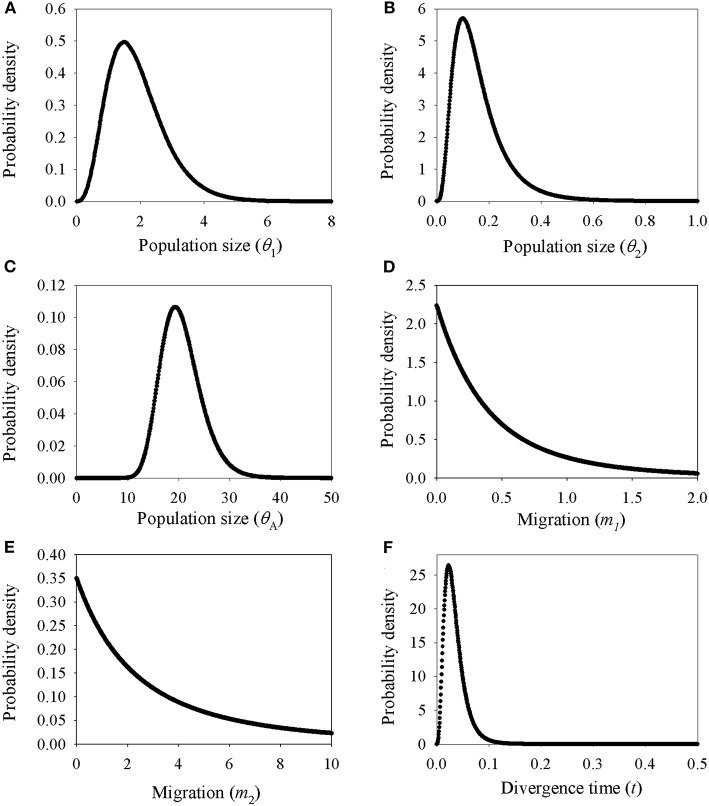
Marginal distribution of posterior probabilities for demographic parameters estimated by IM model. **(A–C)** Marginal distribution of the effective population sizes of *Paeonia delavayi* (θ_1_), *Paeonia ludlowii* (θ_2_), and their ancestral population (θ_A_); **(D,E)** migration rate between *Paeonia delavayi* and *Paeonia ludlowii*; **(F)** the divergence time between *Paeonia delavayi* and *Paeonia ludlowii*.

**Figure 5:**
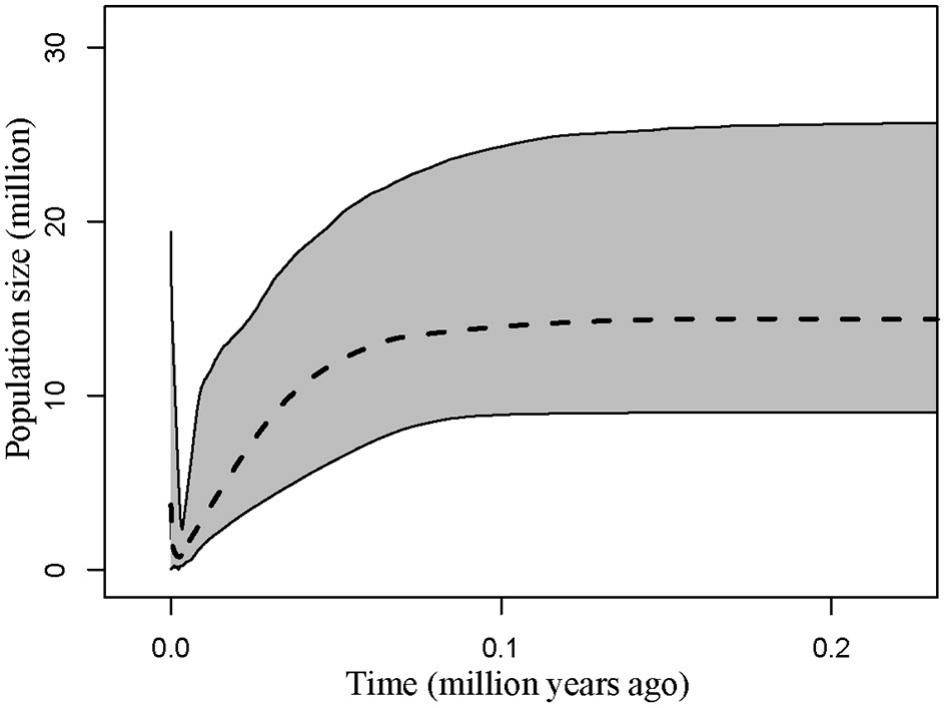
Multilocus extended Bayesian skyline plot for all *Paeonia delavayi* populations. The dotted line is the median posterior effective population size through time; solid lines indicate the 95% highest posterior density interval for each estimate.

Demographic estimates for comparison between *P*. *delavayi* and *P*. *ludlowii* were represented graphically in Figure 4. The effective population size of *P*. *delavayi* (θ_1_ = 1.498) was at least an order of magnitude greater than that found in *P*. *ludlowii* (θ_2_ = 0.100) (Figures 4A,B). Contemporary population sizes in both species were less than those inferred for ancestral populations (θ_A_ = 19.345) (Figure 4C), suggesting population declines from historical levels. The posterior probability for m_1_ and m_2_ rose asymptotically as both parameters approached zero, suggesting that *P*. *delavayi* and *P*. *ludlowii* were isolated and not connected by gene flow (*m*_1_ = *m*_2_ = 0) (Figures 4D,E). The posterior probability of *t* was peaked at about 0.023 (HPDI = 0.006–0.061) (Figure 4F), and after being scaled by the substitution rate, the divergence time was ca. 26 thousand years ago (ka) (HPDI = 7.3–71 ka), that is, the Late Quaternary. Extended Bayesian skyline plots for all individuals had a dramatic signature of decline gradually since roughly 70 ka. Although there is a signature of very recent growth, EBSP for individual samples of *P*. *delavayi* indicated evidence of historical larger *N*e, contrasting with the current estimate of *N*e (Figure 5). In DIYABC, scenario 2 had the highest posterior probability: 0.374 (95% CI: 0, 0.798) for direct estimation; 0.395 (95% CI: 0.365, 0.425) for logistic regression tests. Ninety-five percent CI for this model did not overlap with those obtained for the other scenarios (Supplementary Figure 4). In addition, model checking analysis showed that in scenario 2, the observed data point was centered around the cluster of points for the simulated data based on the posterior distributions (Supplementary Figure 5). Scenario 2 described a history that *P*. *ludlowii* was most likely derived from *P*. *delavayi* and established its current distribution through recent expansion.

## Discussion

### Relationship Between *P*. *delavayi* and *P*. *ludlowii*

The sister relationship between *P*. *ludlowii* and *P*. *delavayi* has been proved in the previous study (Zhou et al., 2014); however, these phylogenies were inferred from small sampling sizes of the extant populations of *P*. *ludlowii* and *P*. *delavayi*. In this study, the nuclear gene tree showed that *P*. *ludlowii* and *P*. *delavayi* were two independent clades with strong support, and both were a sister group (Figure 3). It is the first time to support the current taxonomic treatment that *P*. *ludlowii* and *P*. *delavayi* are two different species by samplings population genetic samples. However, our new result revealed that the clade of *P*. *ludlowii* was nested within *P*. *delavayi* forming a clade with weak support based on the tree constructed by cpDNA sequences (Supplementary Figure 3), further confirming the low level of chloroplast genome divergences (Table 1), and progenitor–derivative relationship (Zhou et al., 2014; Zhang J. M. et al., 2018).

This incongruence could have been a result of lineage sorting and/or natural hybridization and the low number of genes (Jakob and Blattner, 2006; Abbott et al., 2013). However, the genetic footprints of hybridization were not detected from the cpDNA polymorphisms, and the demographic analyses also showed that the two species diverged without the presence of gene flow (Figure 4). Further, the haplotypes were all species-specific and not shared across the two species within the whole distribution range (Figure 1 and Supplementary Figure 1). Therefore, these results together suggested that incomplete lineage sorting may be the reasonable explanation for the gene tree heterogeneity. This is particularly the case if coalescence is predicted to need much more time that has elapsed since the relevant speciation event and more likely involved in species with large *N*e (Naciri and Linder, 2015). Moreover, alternatively the discrepancy suggests that the two species might diverge recently (see discussion below).

### Late Quaternary Speciation of *P*. *ludlowii*

Whether fast progenitor-derivative speciation is promoted by the complete termination of gene flow through geographic isolation or ecological and mating selection pressure reducing interspecific gene flow is a matter of debate (Coyne and Orr, 2004; Crawford, 2010). The general expectation of geographic isolation as the only or main cause of genetic divergence among spatially isolated populations implies that allopatric speciation predominates in HHM (Yu et al., 2019). In subsect. *Delavayanae*, the evidence of IBE revealed from the neutral nuclear gene implies that ecological processes are also likely responsible for the genetic divergence between *P*. *delavayi* and *P*. *ludlowii*. The Quaternary climatic oscillations in HHM regions have already been described in facilitating speciation and diversification (see review by Wen et al., 2014). However, only a few recent researches have revealed that ecological factors have promoted genetic divergence between sister species in the HHM, such as *Roscoea cautleoides* derived from *R*. *humeana* due to the Quaternary glacial and interglacial fluctuations (Zhao et al., 2016), and climatic differentiation driving peripheral speciation of *Sophora moorcroftiana* in the Yarlung Zangbo River (Cheng et al., 2017). Similar to these species pairs, the progenitor–derivative relationship was further confirmed by the ABC method. IMa and ABC analyses indicated that the divergence between *P*. *delavayi* and *P*. *ludlowii* is estimated to take place during the last glaciation (Figure 3 and Supplementary Figure 4), which started from 70 ka and continued until the end of the LGM in the QTP, resulting in the decrease of temperature and precipitation (Shi, 2002; Zheng et al., 2002). Such climatic changes could have triggered the divergence within subsect. *Delavayanae*. Therefore, ecological selection during the glaciation could have profound effects in speciation of *P*. *ludlowii*, and niche differentiation between the two species provides another evidence for divergence of locally populations (Zhang J. M. et al., 2018). However, except for the IBE associated with genetic variation, significant IBD was also revealed within subsect. *Delavayanae*, indicating a combination of allopatric and ecological speciation (Schluter, 2001).

The glacial refugia could support speciation and endemism by providing opportunities for diverging populations to become more specialized and evolve into incipient species (Dufresnes et al., 2020). This is likely the case for the Yarlung Zangbo Valley, which acted as micro-refugia for quite a few species during the glaciations (Liu et al., 2014; Yu et al., 2019). Therefore, it is probably that ancestor-like *P*. *delavayi* of *P*. *ludlowii* retreated to micro-refugia such as Yarlung Zangbo Valley in QTP when their distribution areas became unsuitable due to the climate change during the glaciations, and divergent selection led to local adaptation to the climatic conditions. Such adaptation is also reflected by the morphological variation in comparison to *P*. *delavayi*, such as a height of 1.5–3.5 m, pure yellow petals, and 1–2 carpels. Environmental gradients associated with altitude can facilitate niche divergence (Korner, 2007). As a species endemic to QTP, *P*. *ludlowii* occupies semi-arid and semi-humid habitat at higher altitudes in comparison to *P*. *delavayi*. Such adaptation to high elevation in contrast to the ancestor was also reported in other species such as *Pinus densata* (Mao and Wang, 2011) and *Rana kukunoris* (Zhou et al., 2012) distributed in the HHM and adjacent regions. The lines of evidence of low-level cpDNA genetic diversity and one nuclear haplotype fixed in all populations, as well as lots of mutation steps separated the cpDNA clade (comprising C17, C27, and C30) and nuclear haplotypes (A22) from the closest haplotypes (Figure 1 and Supplementary Figure 1), indicated a scenario of *in situ* persistence of *P*. *ludlowii* accompanied by serious bottlenecks and genetic drift. The evidence for IMa analyses also supports no interspecific genetic admixture (Figure 4).

### Genetic Differentiation and Demographic History Within *P*. *delavayi*

Although the lines of evidence inferred from the demographic history analyses dismiss the possibility of speciation in *P*. *ludlowii* directly triggered by the mountain uplifts in HHM, the long-term geographic isolation of the ancestor *P*. *delavayi* populations associated with these geological processes cannot be precluded. Our analyses showed that coalescence of all sampled cpDNA haplotypes could date to the Late Miocene (95% HPD: 7.14–15.06 Mya), suggesting that *P*. *delavayi* has a long evolutionary history and both complex tectonic events (e.g., the final extension of the QTP during the Late Miocene and the Pliocene, particularly at its eastern edge encompassing HDM, and major uplift after the Miocene reaching peak elevation shortly before the Late Pliocene; Favre et al., 2015) and Pleistocene climate changes presumably contributed to population isolation and thus to allopatric genetic differentiation between isolated populations. Geographic isolation has been proposed as a major driving force of speciation and rapid diversity or lineage diversification for *Paeonia* species such as *P*. *rockii* (Sang et al., 1997; Yuan et al., 2012), and lineage divergence driven by topographically induced allopatric divergence has been also found in other species in HHM (see review by Liu et al., 2017). Similarly, the stronger pattern of IBD found in *P*. *delavayi* most likely reflects that isolation by distance may be the major driver of genetic variation patterns. Strong barriers of parallel north–south-oriented valleys (the Yangzi, the Mekong, and the Salween valleys) surrounded by high peaks (such as Nushan and Gaoligongshan) resulted from HHM uplift events which favored deep allopatric divergences, and perhaps lineage persistence during the ensuing Pleistocene climatic fluctuation in “refugia within refugia” of *P*. *delavayi*.

However, no reliable distinguishable lineage within *P*. *delavayi* were obtained and the precise divergence time for intraspecific lineages could not be estimated from the present genetic datasets, but the fixed private haplotypes in most populations and very low levels of admixture in the nuclear gene indicated that *P*. *delavayi* is on the way of speciation (incipient speciation) (Liu, 2016; Gao et al., 2019). Particularly, from the results of STRUCTURE (Figure 2), the populations distributed in Yunnan Plateau (yellow or yellow green petals), Eryuan (yellow green petals), and Tibet clearly represented the most differentiated lineages in all analyses and may be in more active speciation. Further, almost all of the bioclimatic variables among these three regions (YP, HDM, and EH) were significantly distinct (Supplementary Table 5), which suggests that the difference in morphology of *P*. *delavayi* among these regions is also likely the result of long-term adaptation to the climatic heterogeneity, irrespective of geographic distances.

In addition, we found a large number of missing intermediates predicted by the network, indicating that serious bottleneck of *P*. *delavayi* must have taken place resulting in the loss of chloroplast haplotypes. This result is concordant with the coalescent analyses, in which the estimated population size was obviously smaller than that of the ancestral population (Figure 4). According to the results of EBSP analyses, the most dramatic decline in effective population size occurred since roughly 70 ka, the major glaciation period on the QTP (Zheng et al., 2002; Figure 5). This result was also consistent with previous ecological niche models. According to the niche distribution modeling, temperature is the critical factor impact on the distribution of *P*. *delavayi* (Zhang J. M. et al., 2018; Zhang K. L. et al., 2018). It is suggested that temperature during the last glacial maximum (LGM) was 6–9°C lower than that in today on the QTP. A cooling climate is thought to push species to lower elevations or latitudes (Davis and Shaw, 2001), and if the temperature reduced too much then the species may be completely extirpated from mountains. As a result, it is possible that greater bottleneck or extinction could have occurred in *P*. *delavayi* during the Late Pleistocene, because the east–west orientation of valleys would have inhibited migration toward southern ice-free areas during glacial cycles, compared with the HDM region, and similar patterns of climate-related bottleneck or extinction have been found in other plants distributed in HHM (Xing and Ree, 2017). Besides, recent anthropogenic pressures on *P*. *delavayi* populations and habitat might have further intensified genetic bottlenecks within the last decades (Hong et al., 2017). Although there is a signature of very recent growth, EBSP for individual samples of *P*. *delavayi* showed evidence of historical larger *N*e, contrasting with current estimate of *N*e, suggesting severe population decline. Even so, a relatively large effective population size was still maintained in *P*. *delavayi*, which is reflected by higher or close levels of genetic diversity (cpDNA: Hd = 0.958, π = 0.003; nDNA: Hd = 0.933, π = 0.006), compared with those of congeneric species including *P*. *jishanensis, P*. *qiui*, and *P*. *rockii* or other shrubs distributed in HHM such as *S*. *moorcroftiana* (Cheng et al., 2017), *Spiraea alpina* (Khan et al., 2018), and *Rosa sericea* (Gao et al., 2019).

## Conclusions

Our phylogenetic analyses support the current taxonomic treatment that *P*. *ludlowii* and *P*. *delavayi* are two different species. We found evidence for ecological divergence with allopatric speciation as the mode of speciation for *P*. *ludlowii*, but the genetic divergence within *P*. *delavayi* was mainly attributed to geographical isolation. In addition, our integrative analyses highlight the role of climatic change during the Late Quaternary in the speciation and population dynamics of subsect. *Delavayanae*. For *P*. *delavayi*, which is under active speciation, efforts need to be undertaken to obtain more precise estimates with multiple loci from the genome sequencing approach and perhaps the parameter-rich evolutionary model to resolve the complicated evolutionary history and cryptic speciation. Overall, our study bears important implications for our understanding of the mechanism of speciation for widespread and endemic plants in HHM.

## Data Availability Statement

The datasets presented in this study can be found in online repositories. The names of the repository/repositories and accession number(s) can be found in the article/Supplementary Material.

## Author Contributions

Y-JZ and XG planned and designed the research. Y-JZ, G-SY, BT, and Y-ZP conducted the field work and drew the figures. Y-JZ and G-SY performed the experiments and analyzed the data. Y-JZ and XG wrote the manuscript. All authors contributed to the article and approved the submitted version.

## Conflict of Interest

The authors declare that the research was conducted in the absence of any commercial or financial relationships that could be construed as a potential conflict of interest.
