# Supplementary material for: Climatic Refugia and Geographical Isolation Contribute to the Speciation and Genetic Divergence in Himalayan-Hengduan Tree Peonies (Paeonia delavayi and Paeonia ludlowii)
Source: Front Genet. 2021 Jan 27;11:595334. doi: 10.3389/fgene.2020.595334 (PMC7874331; doi:10.3389/fgene.2020.595334)
Supplement: Supplementary file 1 [file Data_Sheet_1.pdf]

Supplementary Material

Climatic refugia and geographical isolation contribute to the speciation and genetic divergence in Himalayan-Hengduan tree peonies (*Paeonia delavayi* and *Paeonia ludlowii*)

Yu-Juan Zhao, Gen-Shen Yin, Yue- Zhi Pan, Bo Tian, Xun Gong

Tables and Figures

Table 1 | Collection localities, coordinates, sample size and recovered haplotypes based on chloroplast and nuclear data set from populations of *Paeonia delavayi* and *Paeonia ludlowii*.

Code	Population	Longitude (E °)	Latitude (N °)	Elevation (m)	Sample size	cpDNA Haplotypes (number of sequences)	Nuclear Haplotypes (number of sequences)
Paeonia delavayi							
Yunnan Plateau							
Pd1	Kunming, Yunnan	102.9	24.75	2350	10	C5 (8), C6 (2)	A5 (17), A6 (3)
Pd2	Kunming,Yunnan	102.62	24.95	1980	10	C14 (10)	A5 (20)
Pd3	Kunming, Yunnan	103.51	24.82	2100	10	C29 (10)	A31 (20)
Hengduan Mountians							
Pd4	Dali,Yunnan	100.02	25.91	2570	10	C13 (10)	A4 (10), A15 (10)
Pd5	Eryuan, Yunnan	99.83	26.01	3415	10	C10 (10)	A10 (20)
Pd6	Lijiang, Yunnan	100.02	26.79	3210	10	C16 (10)	A3 (8), A21 (12)
Pd7	Lijiang, Yunnan	100.21	27.01	3600	10	C18 (10)	A3 (8), A4 (6), A23 (2), A24 (4)
Pd8	Lijiang, Yunnan	100.25	27.05	3050	10	C11 (10)	A3 (10), A4 (6), A11 (4)
Pd9	Lijiang, Yunnan	100.18	27.01	3300-3500	10	C11 (10)	A3 (4), A18 (3), A19 (9), A20 (4)
Pd10	Lijiang, Yunnan	100.26	27.2	2695	10	C22 (10)	A3 (17), A4 (2), A23 (1)

Pd11	Lijiang, Yunnan	99.97	27.33	2605	10	C3 (8), C4 (2)	A2 (12), A3 (4), A4 (4)
Pd12	Xianggelila, Yunnan	99.95	27.4	2705	10	C31 (10)	A2 (10), A4 (10)
Pd13	Xianggelila, Yunnan	99.6	27.91	3296	10	C24 (9), C25 (1)	A3 (2), A4 (6), A30 (12)
Pd14	Xianggelila, Yunnan	100.01	27.6	2410	10	C28 (10)	A14(6), A24(14)
Pd15	Xianggelila, Yunnan	99.53	28.01	3160	10	C24 (7), C26 (3)	A4(16), A23(4)
Pd16	Xianggelila, Yunnan	99.73	28.22	2950	10	C12 (10)	A12(10), A13(6), A14(4)
Pd17	Weixi, Yunnan	98.83	27.67	3695	10	C15 (10)	A4 (11), A16 (6), A17 (3)
Pd18	Weixi, Yunnan	99.01	27.7	3450	10	C9 (10)	A9 (10), A32 (10)
Pd19	Weixi, Yunnan	99.01	27.86	2850	10	C32 (10)	A4 (4), A16 (6), A17 (4), A33 (6)
Pd20	Deqin, Yunnan	99.17	28.17	2962	10	C7 (6), C8 (4)	A7 (14), A8 (6)
Pd21	Deqin, Yunnan	98.82	28.46	2730	10	C9 (10)	A9 (20)
Pd22	Deqin, Yunnan	98.81	28.43	3105	10	C9 (10)	A9 (20)
Pd23	Lijiang, Yunnan	100.7	27.65	2685	10	C23 (10)	A24 (20)
Pd24	Yanyuan, Sichuan	100.76	27.75	2750	10	C33 (10)	A4 (14), A34 (6)
Pd25	Muli, Sichuan	100.8	28.01	3050	10	C21 (10)	A25 (20)
Pd26	Muli, Sichuan	100.85	28.27	2590	10	C19 (10)	A25 (16), A26 (2), A27 (2)

Pd27	Muli, Sichuan	100.9	28.5	3480	10	C20 (10)	A28 (12), A29 (8)
East Himalaya							
Pd28	Chayu, Tibet	97.53	28.78	2800	10	C2 (10)	A1 (20)
Pd29	Chayu, Tibet	97.4	28.98	2850	10	C1 (10)	A1 (20)
Pd30	Cahyu, Tibet	97.2	29.15	3400	7	C2 (7)	A1 (20)
Pd31	Bomi, Tibet	95.6	29.9	2906	10	C2 (10)	A1 (14)
Paeonia ludlowii							
East Himalaya							
Pl1	Mainling, Tibet	94.45	29.46	3100	8	C17 (10)	A22 (20)
Pl2	Mainling, Tibet	94.38	29.34	2975	10	C30 (10)	A22 (20)
Pl3	Mainling, Tibet	94.15	29.19	2970	10	C17 (4), C27 (4)	A22 (16)

Table 2 | The chloroplast DNA fragment, nuclear gene information and list of the primers used in this study.

fragment or locus	PCR primers	Reference
*trn*L-*trn*F	F: CGA AAT CGG TAG ACG CTA CG	(Taberlet et al., 1991)
	R: ATT TGA ACT GGT GAC ACG AG	
*rps*16-*trn*K	F: AAA GTG GGT TTT TAT GAT CC	(Shaw et al., 2007)
	R: TTA AAA GCC GAG TAC TCT ACC	
*trn*H-*psb*A	F: CGC GCA TGG TGG ATT CAC AAA TC	(Dong et al., 2012)
	R: TGC ATG GTT CCT TGG TAA CTT C	
ATP-dependent Lon protease	F: TAA CTT CGA GCG TTG TCT	(Zhou et al., 2014)
	R: CTG CTG GTG CTG TAC CTA AA	

Table 3 | Chloroplast DNA sequence polymorphism detected in *Paeonia* subsect. *Delavayanae* at three chrolopast DNA fragments and identifying 33 chlorotypes (C1−33).

Haplotypes	Nucleotide position																														
			-																			1	1	1	1	1	1	1	1	1	1	1
	1	2	2	3	3	3	4	5	5	6	6	6	6	6	7	7	7	8	8	9	9	1	1	1	1	1	1	1	1	1	2	2
	3	1	3	3	3	6	4	4	7	3	4	6	7	9	0	3	5	6	9	2	9	2	6	7	7	8	8	9	9	9	0	3
	4	7	1	1	3	1	8	2	9	4	1	4	3	4	3	3	9	0	8	2	1	2	5	1	6	5	6	0	1	3	1	2
C1	C	G	1[a]	A	G	T	T	A	1[b]	C	T	G	C	C	G	A	1[c]	A	A	A	A	C	G	A	G	A	1[d]	A	1[e]	1[f]	1[g]	A
C2	.	.	1[a]	.	.	.	.	.	1[b]	.	.	.	.	.	.	.	1[c]	.	.	.	.	.	.	.	.	.	1[d]	.	1[e]	1[f]	1[g]	.
C3	.	.	1[a]	.	.	.	.	.	1[b]	.	G	.	.	.	.	.	1[c]	.	G	.	.	.	.	C	T	.	1[d]	.	1[e]	1[f]	1[g]	.
C4	.	.	1[a]	.	.	.	.	.	1[b]	.	G	.	.	.	.	.	1[c]	.	G	.	.	.	.	C	T	.	1[d]	.	1[e]	1[f]	1[g]	.
C5	-	.	1[a]	.	.	.	.	.	0	T	G	.	.	.	.	.	1[c]	.	.	.	.	T	C	.	T	.	1[d]	.	1[e]	1[f]	1[g]	G
C6	-	.	1[a]	.	.	.	.	.	0	T	G	.	.	.	.	.	1[c]	.	.	.	.	T	C	.	T	.	1[d]	.	1[e]	1[f]	1[g]	G
C7	.	.	1[a]	.	.	.	.	.	1[b]	.	G	.	.	.	.	.	1[c]	.	.	.	.	.	.	.	T	.	0	-	0	1[f]	1[g]	.
C8	.	.	1[a]	.	.	.	.	.	1[b]	.	G	.	.	.	.	.	1[c]	.	.	.	.	.	.	.	T	.	0	-	0	1[f]	1[g]	.
C9	.	.	1[a]	.	.	.	.	.	1[b]	.	G	.	.	.	.	.	1[c]	.	.	.	.	.	.	.	T	.	1[d]	.	1[e]	1[f]	1[g]	.
C10	.	.	1[a]	.	.	.	.	.	1[b]	.	G	.	.	.	.	.	1[c]	.	.	.	.	.	.	.	T	.	1[d]	.	1[e]	1[f]	1[g]	.

C11	.	.	1[a]	.	.	.	.	C	1[b]	.	G	.	.	.	.	.	1[c]	.	.	.	.	.	.	C	T	.	1[d]	.	1[e]	1[f]	1[g]	.
C12	.	.	1[a]	.	.	.	.	.	0	.	G	.	.	.	.	.	1[c]	.	.	G	.	.	C	.	T	.	1[d]	.	1[e]	1[f]	1[g]	G
C13	.	.	1[a]	.	T	.	.	.	0	.	G	.	.	.	.	C	1[c]	.	.	.	.	.	C		T	-	0	-	0	0	0	-
C14	-	.	1[a]	.		.	.	.	0	T	G	.	.	.	.	.	1[c]	.	.	.	.	.	C	.	T	.	1[d]	.	1[e]	1[f]	1[g]	G
C15	.	.	1[a]	.	T	.	.	.	0	.	G	A	.	.	.	.	1[c]	.	.	.	.	.	C	.	T	-	0	-	0	0	0	-
C16	.	.	1[a]	.	.	.	.	.	1[b]	.	G	.	.	.	.	.	1[c]	.	.	.	.	.	.	C	T	.	1[d]	.	1[e]	1[f]	1[g]	.
C17	.	.	1[a]	.	.	.	G	.	1[b]	.	G	.	.	A	.	.	1[c]	.	.	.	.	.	.	.	T	.	1[d]	.	1[e]	0	1[g]	G
C18	.	.	1[a]	.	.	.	.	C	1[b]	.	G	.	.	.	.	.	1[c]	.	.	.	-	.	.	C	T	.	1[d]	.	1[e]	1[f]	1[g]	.
C19	.	.	1[a]	.	.	.	.	.	0	.	G	.	.	.	.	.	1[c]	G	.	.	.	.	C	.		.	1[d]	C	1[e]	1[f]	1[g]	G
C20	.	.	1[a]	.	.	.	.	.	0	.	G	.	.	.	.	.	1[c]	G	.	.	.	.	C	.	T	.	1[d]	.	1[e]	1[f]	1[g]	G
C21	.	.	1[a]	.	.	.	.	.	0	.	G	.	.	.	.	.	1[c]	G	.	.	.	.	C	.	T	.	1[d]	.	1[e]	1[f]	1[g]	G
C22	.	.	1[a]	.	.	G	.	.	1[b]	.	G	.	.	.	.	.	1[c]	.	.	.	.	.	.	C	T	.	1[d]	.	1[e]	1[f]	1[g]	.
C23	.	-	1[a]	.	.	.	.	.	1[b]	.	G	.	A	.	T	.	0	.	.	.	.	.	.	.	T	.	1[d]	.	1[e]	1[f]	1[g]	G
C24	.	.	0	.	.	.	.	.	0	.	G	.	.	.	.	.	1[c]	.	.	.	.	.	C	.	T	.	1[d]	.	1[e]	1[f]	1[g]	G
C25	.	.	0	.	.	.	.	.	0	.	G	.	.	.	.	.	1[c]	.	.	.	.	.	C	.	T	.	1[d]	.	1[e]	1[f]	1[g]	G
C26	.	.	1[a]	.	.	.	.	.	0	.	G	.	.	.	.	.	1[c]	.	.	.	.	.	C	.	T	.	1[d]	.	1[e]	1[f]	1[g]	G

C27	.	.	1[a]	.	.	G	.	.	0	.	G	.	.	A	.	.	1[c]	.	.	.	.	.	C	.	T	.	1[d]	.	1[e]	0	1[g]	G
C28	.	.	1[a]	C	.	.	.	.	1[b]	.	G	.	.	.	.	.	1[c]	.	.	.	.	.	.	C	T	.	1[d]	.	1[e]	1[f]	1[g]	.
C29	-	.	1[a]	.	.	.	.	.	0	T	G	.	.	.	.	.	1[c]	.	.	G	.	T	C	.	T	.	1[d]	.	1[e]	1[f]	1[g]	G
C30	.	.	1[a]	.	.	G	.	.	0	.	G	.	.	A	.	.	1[c]	.	.	.	.	.	C	.	T	.	1[d]	.	1[e]	0	1[g]	G
C31	.	.	1[a]	.	.	.	.	.	1[b]	.	G	.	.	.	.	.	1[c]	.	.	.	.	.	.	C	T	.	1[d]	.	1[e]	1[f]	1[g]	.
C32	.	.	1[a]	.	.	.	.	.	0	.	G	.	.	.	.	.	1[c]	.	.	.	-	.	C	.	T	-	0	-	0	0	0	-
C33	.	-	1[a]	.	.	.	.	.	1[b]	.	G	.	A	.	T	.	0	.	.	.	.	.	.	.	T	.	1[d]	.	1[e]	1[f]	1[g]	G

Continued

Haplotypes	Nucleotide position																												
	1	1	1	1	1	1	1	1	1	1	1	1	1	1	1	1	1	1	1	1	1	1	1	1	2	2	2	2	2	2
	2	2	2	3	3	3	3	4	4	5	5	5	6	6	6	7	7	7	8	8	9	9	9	9	0	0	0	0	0	4
	3	6	6	1	1	2	4	5	7	2	4	9	3	5	6	1	2	5	2	3	1	5	9	9	0	1	1	3	4	0
	3	2	3	6	7	2	3	9	9	5	6	2	4	4	9	0	5	0	3	8	1	1	6	7	1	0	1	7	7	3
C1	1^h	T	1^i	A	1^j	A	C	A	0	A	A	T	T	G	T	G	T	G	0	G	0	A	-	T	T	G	1^n	G	2^a	G
C2	1^h	.	1^i	.	1^j	.	.		0	.	.	.	.	.	.	-	.	.	0	.	0	.	-	.	.	G	1^n	.	2^a	.
C3	1^h	.	1^i	.	1^j	.	.		0	.	.	.	.	.	A	-	.	T	1^l	.	0	.	-	.	.	G	1^n	.	2^a	.
C4	1^h	.	1^i	.	1^j	.	.		0	.	.	.	.	.	.	-	.	T	1^l	.	0	.	-	.	.	G	1^n	.	2^a	.
C5	1^h	.	1^i	.	1^j	G	.	C	1^k	.	.	.	.	.	.	-	.	.	0	T	0	.	A	.	.	G	1^n	.	2^b	.
C6	1^h	.	1^i	.	1^j	G	.	C	1^k	.	.	.	.	.	A	-	.	.	0	T	0	.	A	.	.	G	1^n	.	2^b	.
C7	1^h	.	1^i	.	1^j	.	.		0	.	.	.	.	.	.	-	.	.	0	.	0	.	-	.	.	G	1^n	.	2^a	.
C8	1^h	G	1^i	.	1^j	.	.		0	.	.	.	.	.	.	-	.	.	0	.	0	.	-	.	.	G	1^n	.	2^a	.
C9	1^h	.	1^i	.	1^j	.	.		0	.	.	.	.	.	.	-	.	.	0	.	0	.	-	.	.	G	1^n	.	2^a	.
C10	1^h	.	1^i	T	1^j	.	.		0	.	T	.	.	.	.	-	.	T	0	.	0	.	-	.	.	G	1^n	.	2^a	.

C11	1^{h}	.	1^{i}	.	1^{j}	.	.		0	.	.	.	.	.	.	-	.	T	0	.	0	.	-	.	.	G	1^{n}	.	2^{a}	.
C12	1^{h}	.	1^{i}	.	1^{j}	G	.	C	1^{k}	G	.	.	.	.	.	-	.	T	0	T	0	.	-	.	.	G	1^{n}	.	2^{a}	.
C13	0	.	0	0	0	G	.	C	1^{k}	.	.	.	.	.	.	-	.	.	0	T	0	.	-	.	.	G	1^{n}	.	2^{a}	.
C14	1^{h}	.	1^{i}	.	1^{j}	G	.	C	1^{k}	.	.	.	.	.	.	-	.	.	0	T	0	.	A	.	.	G	1^{n}	.	2^{a}	.
C15	0	.	0	0	0	G	.	C	1^{k}	.	.	.	.	.	.	-	.	.	0	T	0	.	-	.	.	G	1^{n}	.	2^{a}	.
C16	1^{h}	.	1^{i}	.	1^{j}	.	.		0	.	.	.	.	.	.	-	.	T	0	.	0	.	-	.	.	G	1^{n}	.	2^{b}	.
C17	1^{h}	.	1^{i}	.	1^{j}	G	.	C	1^{k}	.	.	.	.	.	.	-	.	.	0	T	0	.	-	.	-	T	1^{n}	.	2^{a}	.
C18	1^{h}	.	1^{i}	.	1^{j}	.	.		0	.	.	.	.	.	.	-	.	T	0	.	0	.	-	.	.	G	1^{n}	.	2^{a}	.
C19	1^{h}	.	1^{i}	.	1^{j}	G	.	C	1^{k}	.	.	G	.	.	.	-	.	T	0	T	0	.	-	.	-	-	0	.	2^{a}	.
C20	1^{h}	.	1^{i}	.	1^{j}	G	.	C	1^{k}	.	.	G	.	.	.	-	.	T	0	T	0	.	-	.	-	-	0	.	2^{a}	.
C21	1^{h}	.	1^{i}	.	1^{j}	G	.	C	1^{k}	.	.	G	.	.	.	-	.	.	0	T	0	.	-	.	-	-	0	.	2^{a}	.
C22	1^{h}	.	1^{i}	.	1^{j}	.	.		0	.	.	.	.	.	.	-	.	T	0	.	0	.	-	.	.	G	1^{n}	.	2^{a}	.
C23	1^{h}	.	1^{i}	.	1^{j}	.	.		0	.	.	.	.	A	.	-	.	T	0	T	0	.	-	.	.	G	1^{n}	.	2^{a}	A
C24	1^{h}	.	1^{i}	.	1^{j}	G	.	C	1^{k}	G	.	.	G	.	.	-	.	T	0	T	0	.	-	.	.	T	1^{n}	A	2^{a}	.
C25	1^{h}	.	1^{i}	.	1^{j}	G	.	C	1^{k}	G	.	.	G	.	.	-	.	T	0	.	0	.	-	.	.	G	1^{n}	A	2^{a}	.
C26	1^{h}	.	1^{i}	.	1^{j}	G	.	C	1^{k}	G	.	.	G	.	.	-	.	T	0	T	0	.	-	.	.	T	1^{n}	A	2^{a}	.

C27	1^{h}	.	1^{i}	.	1^{j}	G	.	C	1^{k}	.	.	.	.	.	.	-	.	.	0	.	0	.	-	.	.	G	1^{n}	.	2^{a}	.
C28	1^{h}	.	1^{i}	.	1^{j}	.	.		0	.	.	.	.	.	.	-	.	.	0	.	0	.	-	.	.	G	1^{n}	.	2^{a}	.
C29	1^{h}	.	1^{i}	.	1^{j}	G	.	C	1^{k}	.	.	.	.	.	.	-	.	.	0	T	0	.	A	.	.	G	1^{n}	.	2^{a}	.
C30	1^{h}	.	1^{i}	.	1^{j}	G	.	C	1^{k}	.	.	.	.	.	.	G	A	.	0	T	0	.	-	.	-	T	1^{n}	.	2^{a}	.
C31	1^{h}	.	1^{i}	.	1^{j}	.	0		0	.	.	.	.	A	.	-	.	T	0	.	0	.	-	C	.	G	1^{n}	.	2^{b}	.
C32	0	.	0	0	0	G	.		0	.	.	.	.	.	.	-	.	.	0	.	0	G	-	.	.	T	1^{n}	.	2^{a}	.
C33	1^{h}	.	1^{i}	.	1^{j}	.	.		0	.	.	.	.	A	.	-	.	T	0	T	1^{m}	.	-	.	.	G	1^{n}	.	2^{a}	.

1^{a}: AAAAT

1^{b}: GTATGGAATG

1^{c}: TTTCGAT

1^{d}: GATT

1^{e}:TA

1^{f}: AGATTATA

1^{g}: GATATAAAATCCCGCGTTCTAGAATAGAACG

1^{h}: CAAAAATCTATTTTATCTTATTGATTTTT

1^{i}: GATATAAATCGAAAAAAATCAAGTGAATGGGAATAAATGACAAAATTGGATTT

1[j]: AAAAT

1[k]: TATT

1[l]: TA

1[m]: AACCAAA

1[n]: AAT

2[a]: CCCCTTGATAGAACAAGGAA

2[b]: TTCCTTGTTCTATCAAGGGG

Table 4 | The nuclear gene polymorphism detected in *Paeonia* subsect. *Delavayanae* identifying 34 (A1−34) haplotypes.

Haplotypes	Nucleotide position																			
							1	1	1	1	1	1	2	2	2	2	2	2	2	2
	3	3	5	6	6	9	2	6	6	7	8	8	2	2	3	3	3	6	7	7
	6	7	4	3	9	3	6	3	9	9	0	5	1	2	6	8	9	6	5	6
A1	A	C	G	T	G	C	T	T	G	A	T	1[a]	C	G	A	G	T	A	C	C
A2	.	.	.	.	.	.	.	.	-	.	.	1[a]	.	.	.	.	.	G	.	.
A3	.	.	.	.	.	.	.	.	.	.	.	1[a]	.	.	.	.	.	.	.	.
A4	.	.	.	.	.	.	.	.	.	.	.	1[a]	.	.	.	.	.	.	.	.
A5	.	.	.	.	.	.	.	.	.	.	.	1[a]	.	.	.	.	.	.	.	.
A6	.	.	.	.	.	.	.	.	.	.	.	1[a]	.	.	.	.	.	.	.	.
A7	.	.	.	.	.	.	.	.	.	G	G	1[a]	.	.	.	.	.	.	.	G
A8	.	.	.	.	.	.	.	.	.	G	.	1[a]	.	.	.	.	.	.	.	G
A9	.	.	.	.	.	.	.	.	.	.	.	0	.	.	.	.	.	.	.	.
A10	.	.	.	.	.	.	.	.	.	.	.	1[a]	.	.	.	.	.	.	.	.
A11	.	.	.	.	.	.	.	.	.	.	.	1[a]	.	.	.	.	.	.	.	.
A12	.	.	C	.	.	.	.	.	.	.	.	1[a]	.	A	.	.	.	.	.	G
A13	.	.	.	.	.	A	.	.	.	G	.	1[a]	.	.	.	.	.	.	.	G
A14	.	.	C	.	.	.	.	.	.	.	.	1[a]	.	A	.	.	.	.	.	G
A15	.	.	.	.	.	.	.	.	.	.	.	1[a]	.	.	.	.	.	.	.	.
A16	.	.	.	.	.	.	.	.	.	.	.	1[a]	.	.	.	.	.	.	.	.
A17	.	.	.	.	.	.	.	.	.	.	.	1[a]	.	.	.	.	.	G	.	.
A18	T	T	.	.	.	.	.	.	.	.	.	1[a]	.	A	.	.	.	.	.	.
A19	T	T	.	.	.	.	.	.	.	.	.	1[a]	.	.	.	.	.	.	.	.
A20	T	T	.	.	.	.	.	.	.	.	.	1[a]	.	A	.	.	.	.	.	.
A21	.	.	.	.	A	.	C	.	.	.	.	1[a]	.	.	.	.	.	.	.	.
A22	.	.	.	A	.	.	.	.	.	G	.	1[a]	T	.	.	A	A	.	-	G
A23	.	.	.	.	.	.	.	.	.	.	.	1[a]	.	A	.	.	.	.	.	.
A24	.	.	.	.	.	.	.	.	.	.	.	1[a]	.	A	.	.	.	.	.	G
A25	.	.	.	.	.	.	.	G	.	.	.	1[a]	.	.	.	.	.	.	.	G
A26	.	.	.	.	.	.	.	.	.	.	.	1[a]	.	.	.	.	.	.	.	G
A27	.	.	.	.	.	.	.	G	.	.	.	1[a]	.	.	.	.	.	.	.	G
A28	.	.	.	.	.	.	.	.	.	.	.	1[a]	.	.	G	.	.	.	.	G
A29	.	.	.	.	.	.	.	.	.	.	.	1[a]	.	.	G	.	.	.	.	G
A30	.	.	.	.	.	.	.	.	.	.	.	1[a]	.	.	.	.	.	.	.	.
A31	.	.	.	.	.	.	.	.	.	.	.	1[a]	.	.	.	.	.	.	.	.
A32	.	.	.	.	.	.	.	.	.	.	.	0	.	.	.	.	.	.	.	.
A33	.	.	.	.	.	.	.	.	.	.	.	1[a]	.	.	.	.	.	.	.	.
A34	.	.	.	.	.	.	.	.	.	.	.	1[a]	.	.	.	.	.	.	.	.

Continued

Haplotypes	Nucleotide position																			
	2	2	2	2	3	3	3	3	3	4	4	4	4	4	5	5	5	5	5	5	5
	7	7	9	9	0	1	3	4	9	3	9	9	9	9	0	0	0	1	1	3	4
	7	8	1	3	4	8	5	8	8	4	2	3	5	7	0	8	9	2	7	3	3
A1	G	T	T	A	G	G	T	A	C	C	C	G	T	T	C	C	G	C	C	T	T
A2	.	.	.	.	.	.	.	.	.	.	.	.	.	.	.	.	A	A	.	.	.
A3	.	.	.	.	.	.	.	.	.	.	.	.	.	.	T	.	.	A	.	.	.
A4	.	.	.	.	.	.	.	.	.	.	.	.	.	.	.	.	.	A	.	.	.
A5	A	.	.	.	.	.	.	.	.	.	.	.	.	.	.	.	.	A	.	.	.
A6	A	.	.	.	.	.	.	.	.	.	.	.	.	.	.	.	.	A	.	.	.
A7	C	A	.	.	.	.	C	.	.	.	.	.	.	.	.	.	.	A	.	C	.
A8	.	A	.	.	.	.	C	.	.	.	.	.	.	.	.	.	.	A	.	C	.
A9	.	.	.	.	.	.	.	.	.	.	.	.	.	.	.	.	.	A	.	.	.
A10	.	.	.	.	.	A	.	.	.	.	.	.	.	.	.	.	.	A	.	.	.
A11	.	.	.	.	.	.	.	.	T	.	.	A	.	.	.	.	.	A	.	C	.
A12	C	A	.	.	.	.	.	T	T	.	.	A	.	.	.	.	.	A	.	C	.
A13	C	A	.	.	.	.	C	T	.	.	.	.	C	.	.	.	.	A	.	C	.
A14	C	A	.	.	.	.	.	.	T	.	.	A	.	.	.	.	.	A	.	C	.
A15	.	.	.	.	.	.	.	.	.	.	.	.	.	A	.	.	.	A	.	.	.
A16	.	.	.	.	.	.	.	.	.	.	.	.	.	.	.	.	.	A	T	C	.
A17	.	.	.	.	.	.	.	.	.	.	.	.	.	.	.	.	.	A	.	.	.
A18	.	.	.	.	.	.	.	.	.	.	.	.	.	.	T	.	.	A	.	.	.
A19	.	.	.	.	.	.	.	.	.	.	.	.	.	.	T	.	.	A	.	.	.
A20	.	.	.	.	.	.	.	.	T	.	.	A	.	.	T	.	.	A	.	.	.
A21	.	.	.	.	.	.	.	.	.	.	.	.	.	.	T	.	.	A	.	.	.
A22	C	A	C	.	.	.	.	.	.	.	.	.	.	.	.	T	.	A	.	C	C
A23	.	.	.	.	.	.	.	.	.	.	.	.	.	.	T	.	.	A	.	.	.
A24	C	A	.	.	.	.	.	.	T	.	.	A	.	.	.	.	.	A	.	C	.
A25	C	A	.	.	.	.	.	.	.	.	T	.	.	.	.	.	.	A	.	C	.
A26	C	A	.	.	.	.	.	.	.	.	.	.	.	.	.	.	.	A	.	C	.
A27	C	A	.	.	.	.	.	.	.	.	.	.	.	.	.	.	.	A	.	C	.
A28	C	A	.	.	.	.	.	.	.	.	.	A	.	.	.	.	.	A	.	C	.
A29	C	A	.	.	.	.	.	.	.	.	.	.	.	.	.	.	.	A	.	C	.
A30	.	.	.	T	.	.	.	.	.	.	.	.	.	.	.	.	.	A	.	.	.
A31	A	.	.	.	.	.	.	.	.	.	.	.	.	.	.	.	.	A	.	.	.
A32	.	.	.	.	.	.	.	.	.	.	.	.	.	.	.	.	.	A	T	C	.
A33	.	.	.	.	.	.	.	.	.	.	.	.	.	.	.	.	.	A	.	C	.
A34	.	.	.	.	C	.	.	.	.	.	.	.	.	.	.	.	.	A	.	.	.

1[a]: TACTCGTATTATA

Table 5 | Means, standard errors (SE) and results of Independent-Sample t-test on the 8 Bioclim factors of current periods for the HDM, EH and YP regions of *Paeonia delavayi* surveyed in this study.

Independent-Sample t-test		Bio2	Bio3	Bio4	Bio11	Bio12	Bio14	Bio15	Bio19
HDM	Mean	11.9	47.0	513.74	2.62	893.41	5.11	100.03	22.14
	SE	1.03	2.0	44.17	3.68	127.4	2.41	7.71	10.46
EH	Mean	12.7	44.4	591.8	-1.19	716	3.5	96.08	17.5
	SE	0.64	0.25	26.33	2.60	155.96	1.58	4.01	7.33
YP	Mean	10.5	46.30	457.22	7.05	1032	11.11	93.84	39.66
	SE	0.28	0.61	11.36	2.48	37.77	2.472	3.97	4.47
Comparison on (1-2)									
HDM-EH	*P*	**0.025**	**0**	**0**	**0.002**	**0**	**0.042**	**0.017**	0.175
HDM-YP	*P*	**0**	**0.021**	**0**	**0.001**	**0**	**0**	**0.001**	**0**
EH-YP	*P*	**0**	**0**	**0**	**0**	**0**	**0**	0.239	**0**

Significant level: $P < 0.05$, significant values ($P < 0.05$) are shown in bold. HDM: Hengduan Mountians; EH: East Himalaya; YP: Yunnan Plateau

Table 6 | *trn*L-F, *rps*16_*trn*K, *psb*A-*trn*H and ATP-dependent Lon protease gene haplotypes and Genbank accession numbers for *trn*L-F, *rps*16_*trn*K, *psb*A-*trn*H and ATP-dependent Lon protease gene haplotypes for *Paeonia* subsect. *Delavayanae*.

*trn*L-F		*rps*16_*trn*K		*psb*A-*trn*H		ATP-dependent Lon protease gene	
haplotypes	GenBank	haplotypes	GenBank	haplotypes	GenBank	haplotypes	GenBank
LF_1	MT787349	SK_1	MT814344	HA_1	MT814327	A1	MT814369
LF_2	MT787350	SK_2	MT814345	HA_2	MT814328	A2	MT814370
LF_3	MT787351	SK_3	MT814346	HA_3	MT814329	A3	MT814371
LF_4	MT787352	SK_4	MT814347	HA_4	MT814330	A4	MT814372
LF_5	MT787353	SK_5	MT814348	HA_5	MT814331	A5	MT814373
LF_6	MT787354	SK_6	MT814349	HA_6	MT814332	A6	MT814374
LF_7	MT787355	SK_7	MT814350	HA_7	MT814333	A7	MT814375
LF_8	MT787356	SK_8	MT814351	HA_8	MT814334	A8	MT814376
LF_10	MT787357	SK_9	MT814352	HA_9	MT814335	A9	MT814377
LF_11	MT787358	SK_10	MT814353	HA_10	MT814336	A10	MT814378
LF_12	MT787359	SK_11	MT814354	HA_11	MT814337	A11	MT814379
LF_13	MT787360	SK_12	MT814355	HA_12	MT814338	A12	MT814380

LF_14	MT787361	SK_13	MT814356	HA_13	MT814339	A13	MT814381
LF_15	MT787362	SK_14	MT814357	HA_14	MT814340	A14	MT814382
LF_16	MT787363	SK_15	MT814358	HA_15	MT814341	A15	MT814383
LF_17	MT787364	SK_16	MT814359	HA_16	MT814342	A16	MT814384
LF_18	MT787365	SK_17	MT814360	HA_17	MT814343	A17	MT814385
LF_9	MT787366	SK_18	MT814361			A18	MT814386
		SK_19	MT814362			A19	MT814387
		SK_20	MT814363			A20	MT814388
		SK_21	MT814364			A21	MT814389
		SK_22	MT814365			A22	MT814390
		SK_23	MT814366			A23	MT814391
		SK_24	MT814367			A24	MT814392
		SK_25	MT814368			A25	MT814393
						A26	MT814394
						A27	MT814395
						A28	MT814396

A29	MT814397
A30	MT814398
A31	MT814399
A32	MT814400
A33	MT814401
A34	MT814402

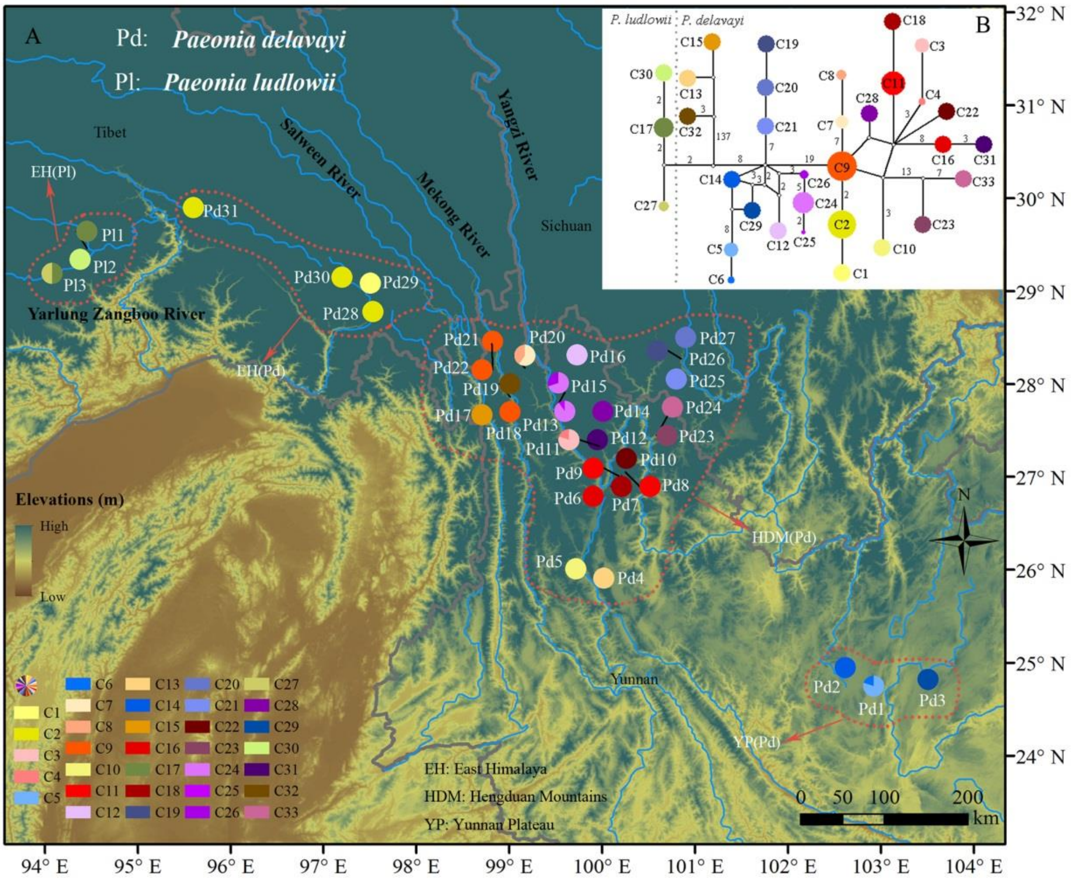

FIGURE 1 | Geographic distribution of the cpDNA haplotypes (**A**) and the median-joining network of 33 cpDNA haplotypes (**B**) detected from 35 *Paeonia* subsect. *Delavayanae* populations in this study. Pie charts division corresponds to the frequency of haplotype per sampling site. Colored dotted line delineates the population division representing significantly genetic divergence. The small open circles represent missing haplotype. The sizes of circles are approximately proportional to sample size. Numbers on branches indicate the number of mutations when branches represent more than one mutation.

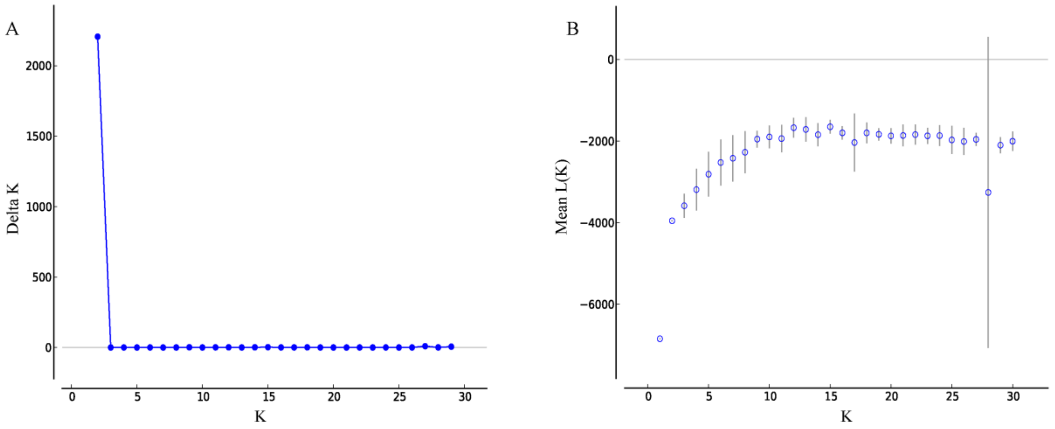

FIGURE 2 | The distribution of Delta K (A) and the mean posterior probability (L(K)) of the data for K =1-30 (10 replicates) (B).

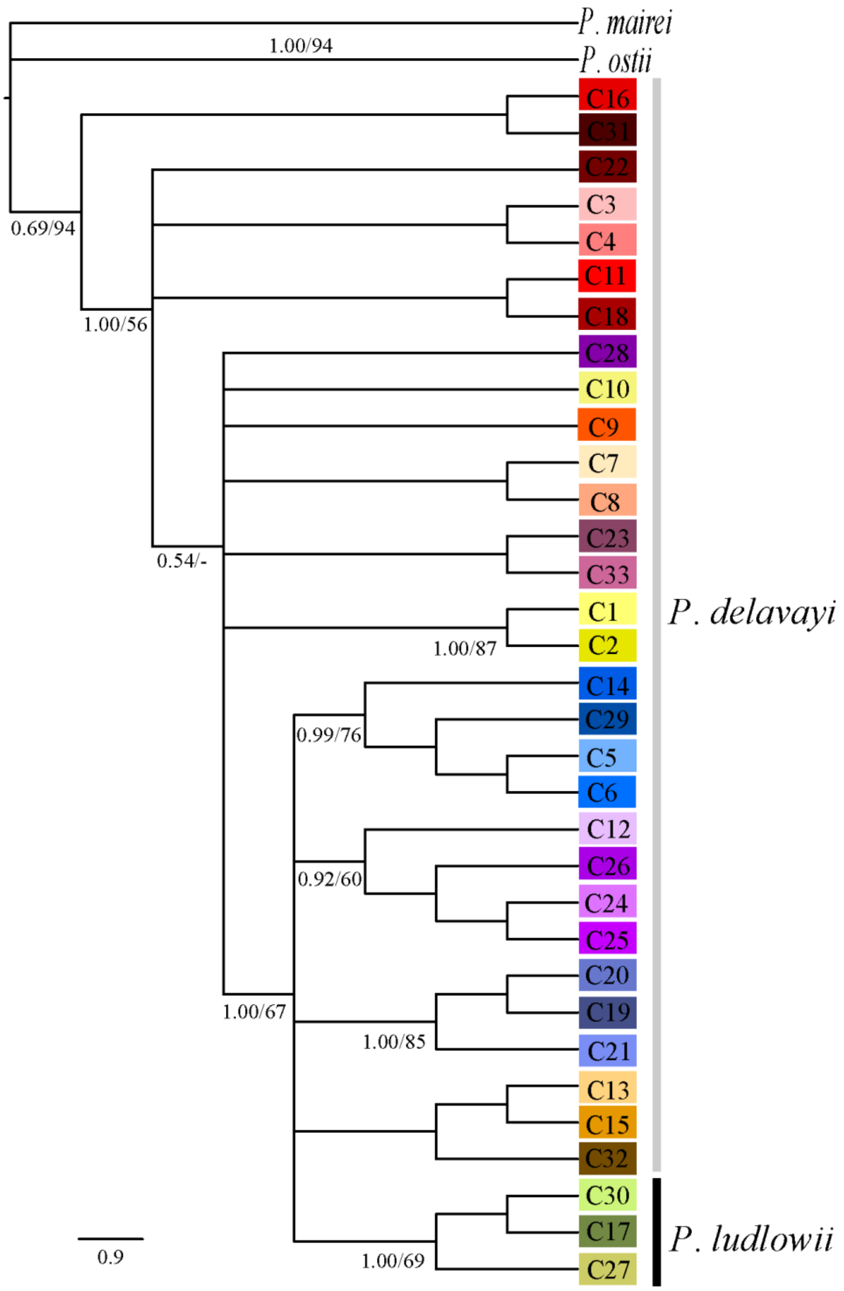

FIGURE 3 | Phylogenetic tree derived from Bayesian inference on haplotypes recovered from three concatenated cpDNA fragemnts (*trn*L-*trn*F, *rps*16-*trn*K, *trn*H-*psb*A) of *Paeonia* subsect. *Delavayanae*. Posterior probabilities and bootstrap support of main clades are shown below the branches.

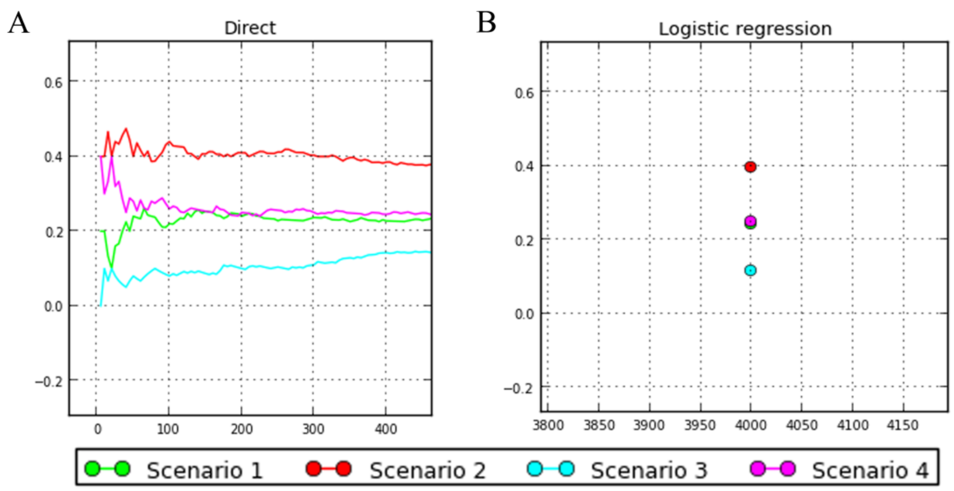

FIGURE 4 | Posterior probabilities of four competing scenarios. (A) obtained using direct estimation of the 500 closest datasets and (B) obtained using logistic regression of 1% of closest datasets.

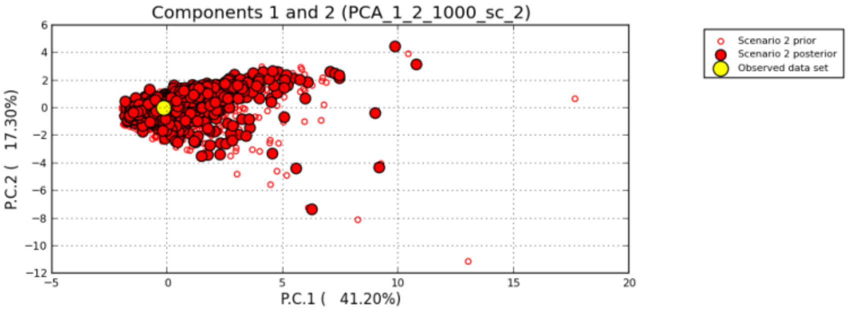

FIGURE 5 | Principle Component Analysis (PCA) obtained by DIYABC.

REFERENCES

Dong, W.P., Liu, J., Yu, J., Wang, L., and Zhou, S.L. (2012). Highly variable chloroplast markers for evaluating plant phylogeny at low taxonomic levels and for DNA barcoding. *PLoS One* 7(4). doi: 10.1371/journal.pone.0035071.

Shaw, J., Lickey, E.B., Schilling, E.E., and Small, R.L. (2007). Comparison of whole chloroplast genome sequences to choose noncoding regions for phylogenetic studies in angiosperms: the tortoise and the hare III. *Am. J. BOT.* 94(3)**,** 275-288. doi: 10.3732/ajb.94.3.275.

Taberlet, P., Gielly, L., Pautou, G., and Bouvet, J. (1991). Universal primers for amplification of three non-coding regions of chloroplast DNA. *Plant Mol. Biol.* 17(5)**,** 1105-1109. doi: 10.1007/bf00037152.

Zhou, S.L., Zou, X.H., Zhou, Z.Q., Liu, J., Xu, C., Yu, J., et al. (2014). Multiple species of wild tree peonies gave rise to the 'king of flowers', *Paeonia suffruticosa* Andrews. *Proc. R. Soc. B-Biol. Sci.* 281(1797). doi: 10.1098/rspb.2014.1687.
